# The Case for Health Reparations

**DOI:** 10.3389/fpubh.2021.664783

**Published:** 2021-07-08

**Authors:** Derek Ross Soled, Avik Chatterjee, Daniele Olveczky, Edwin G. Lindo

**Affiliations:** ^1^Harvard Medical School, Boston, MA, United States; ^2^Harvard Business School, Boston, MA, United States; ^3^School of Medicine, Boston University, Boston, MA, United States; ^4^Boston Medical Center, Boston, MD, United States; ^5^Beth Israel Deaconess Medical Center, Harvard Medical School, Boston, MA, United States; ^6^School of Medicine, University of Washington, Seattle, WA, United States

**Keywords:** reparations for historical injustices, racism and antiracism, health disparities, justice, health policy, historical trauma and historical oppression, medical education, community health

## Abstract

The disproportionate impact of COVID-19 on racially marginalized communities has again raised the issue of what justice in healthcare looks like. Indeed, it is impossible to analyze the meaning of the word justice in the medical context without first discussing the central role of racism in the American scientific and healthcare systems. In summary, we argue that physicians and scientists were the architects and imagination of the racial taxonomy and oppressive machinations upon which this country was founded. This oppressive racial taxonomy reinforced and outlined the myth of biological superiority, which laid the foundation for the political, economic, and systemic power of Whiteness. Therefore, in order to achieve universal racial justice, the nation must first address science and medicine's historical role in scaffolding the structure of racism we bear witness of today. To achieve this objective, one of the first steps, we believe, is for there to be health reparations. More specifically, health reparations should be a central part of establishing racial justice in the United States and not relegated to a secondary status. While other scholars have focused on ways to alleviate healthcare inequities, few have addressed the need for health reparations and the forms they might take. This piece offers the ethical grounds for health reparations and various justice-focused solutions.

## Introduction

The COVID-19 pandemic has exacerbated the inequitable mortality across different racial groups in the United States, highlighting the way in which historical racism in healthcare, housing, employment, and education continues to shape life and death today. Even the rollout of the potentially lifesaving COVID-19 vaccine has been marred by inequity. As physicians seek to gain trust for a wholly untrustworthy healthcare system from historically marginalized and oppressed communities, they have begun to examine medicine's historical role in racist practices. Unfortunately, many of these attempts have fallen short. Consider the Journal of the American Medical Association's podcast in February 2021 that featured two White physicians who questioned whether racism is actually be embedded in society and whether the term racism “might be hurting us” (1). Or as an isolated example, research scholars often cite the Tuskegee experiment, but soul-searching the egregiousness of this one study is woefully insufficient to regain trust, as well as redress past injustices. Indeed, invocations of “Tuskegee” to explain distrust or disparities obscure more telling forms of structural racism (2).

This analysis explores the evolution of medicine's contribution to racial healthcare oppression and its ability to rid itself of the societal scourge. Delving into the history through the present, we show how physicians and scientists created the racial taxonomy and oppression upon which this country was built, a hierarchy that was further honed and wielded so as to maintain and develop the economic, political, and systemic power of Whiteness. This piece then explores a methodology in the form of reparations through which equity could be achieved and the consequences associated therewith. Admittedly, achieving these objectives can be fraught with major challenges, but attainment of these cherished goals would ultimately prove worthwhile. We describe the justification for health reparations and what a blueprint for these policies would look like, with the ultimate goal of achieving long overdue justice for Black people who have suffered because of healthcare's racist past. While this piece focuses on the necessity of health reparations for Black populations, analogous arguments must be made for other marginalized populations, including Native Americans and other communities of color.

### Historical Roots of Racism Stem From Medicine and Research Science

Historically, physicians justified healthcare inequities by claiming that there were genetic and biological differences between races. More specifically, science and medicine have used biological race to support the racist mythical notion that there are “Black” genes, which cause Black individuals to be inferior to White people (3). Examples in which there appears to be some unspecific and undefined biological difference include the essentialist explanation for differences in IQ described in the Bell Curve Intelligence Theory, the Eugenics Movement, and the continued use of biological race in health disparities research, medical guidelines, and standards of care (4).

Prominent examples of when scientists endeavored on their mythical project include the following: (1) Dr. Josiah Nott, a slaveholder and surgeon, from whom came the idea of polygenism, which scientifically supported slavery by arguing that non-European people, particularly, Black people, were a different species; (2) Dr. Samuel Cartwright, who created the pseudo-illness of *drapetomania*, to explain the desire of enslaved people to pursue freedom from bondage as a mental illness, and (3) Dr. Louis Agassiz, a Harvard professor, physician, and another proponent of the theory of polygenism, who championed the American eugenics movement that operationalized the theory that any peoples other than Europeans were physically, socially, and intellectually inferior.

The scientific validation of biological race justified the existence and propagation of racial hierarchy in economics, politics, social structure, and health. This is despite the fact that the myth of biological race had not and has not been supported by genetic findings or linked to genes that affect health. Put plainly, race is a socio-political construct with no biological bearing or determinacy (5–7). In fact, the human genome project revealed that between any two humans, over 90% of genes are identical; the small variation is actually greater between individuals with the same skin color. There are no identifiable continental or racial genomic clusters, yet race has historically—and continues to be—misused as a biological reality in healthcare and biomedical research (8–10). For example, American-born Black people have significantly higher rates of multiple types of cancers compared to sub-Saharan African people, indicating that skin color alone is not a driver of cancer (11). This, along with many other analogous studies, represents a strong argument against race standing in for true physiopathological markers for disease.

Multiple scientists, social scientists, and historians have called for the removal of a biological concept of race in research and practice (12). While the definition of race has been inconsistent for over a century, it nevertheless continues to be used as a taxonomic categorization that is both problematic and harmful (13, 14). As the American Anthropological Association notes, race has always carried more meanings that physical differences—race is “a worldview, a body of prejudgments that distorts our ideas about human differences and group behavior” (15). Racial myths about behavior and physical features have implied that both are genetically determined and interrelated, when in fact this is not, and has never been, the case.

The continued misuse of biological race in medicine reinforces a racial hierarchy and has resulted in disparities in equal access, treatment, and outcomes. Indeed, there is compelling evidence that racism (rather than genes and biology) is a main driver of health inequities. Consider two contemporary examples that demonstrate this point, namely, sickle cell disease and breast cancer. Inequalities in health outcomes for both diseases persist because of inadequate research funding and medical training for “Black” diseases and poor socioeconomic statuses—both driven by historical racism.

Sickle cell disease is labeled as a “Black” disease, despite the fact that people from all different races and ethnicities suffer from it (16–18). This label has caused its research funding and medical education on sickle cell disease to suffer compared to other, historically “White” diseases, such as cystic fibrosis (19). In 1910 physicians labeled sickle cell anemia a Black disease (20). This example of sickle cell plays into the “one-drop rule” that even an ounce of Black blood in one's ancestry was enough to corrupt the genetic purity of Whiteness (21). By the 1950s, although researchers began to argue that the disease was linked to environmental factors, the Journal of the American Medical Association disagreed, claiming “its occurrence depends entirely on the presence of Negro blood, even though in extremely small amounts” (22). The impact of race relations on the disease itself continues to exist today. For instance, sickle cell trait has been cited in dozens of police custody deaths that were ruled accidental or natural, even though the condition is benign on its own (23). Even today, treatment for patients with sickle cell disease lags despite advances in scientific understanding, much in part due to the politics of race and biases (24). Studies have shown that the majority of family physicians feel unqualified to treat sickle cell disease, and many patients who present to the emergency room during acute episodes are denied treatment usually based on faulty assumptions of the Black patients having drug-seeking behavior or a greater pain tolerance, resulting in unnecessary deaths (25, 26).

Second, in the 1930s, medical research used the category of so-called biological race to explain higher rates of breast cancer in Black women compared to White women (27). However, these differences were eliminated once socioeconomic status was controlled (28). Nonetheless, in 2006, medical researchers suggested that increased mortality in Black women with breast cancer was due to an unspecified biological difference, when in reality it was more likely due to unequal treatment and access to mammograms, which were not fully covered by Medicaid (7, 29, 30). By continuing to study and rely on a mythical biological basis for “race” in medicine and science, as opposed to utilizing funds to eliminate barriers tied to socioeconomic status, the racist belief that White people are superior was reaffirmed, and Black individuals were barred from being treated equally by physicians.

In terms of more modern examples, there are many instances in which race has been embedded in the machinery of medicine and in the algorithms applied. For example, Lundy Braun demonstrated how, rooted in racist practices in the antebellum South to demonstrate inferiority of slaves, race is often corrected for in spirometry. These race-based adjustments persist despite the fact that race itself is never universally defined, meaning that differences in lung function are often incorrectly shown to be due to some inherent (or genetic) differences in race (31). Recent reviews have also noted that from cardiology to nephrology to obstetrics to urology, race is used in algorithms to determine organ function, although there are often no racial or ethnic differences that inherently exist (32). Many of these race-adjusted algorithms guide decisions in ways that might direct more resources to White patients than to patients of racial and ethnic minorities; precision technologies and techniques that are supposed to measure biological differences accurate are actually masking racial myths.

Historical mythical conceptions of race as biologic persist today, even in medical education, impacting medical decision making (33). One recent study showed that a substantial number of White laypeople, medical students, and residents hold false beliefs about biological differences between Black and White people and demonstrated that these false beliefs predict racial bias in pain perception and treatment recommendation accuracy (34). This is in part because race is misrepresented in preclinical curricula (35), as well as that over the last 30 years, the world's top medical journals have rarely published scientific articles about the impact of racism on health (36). There are also limits to classroom education on public health and primary care. Misinformation about biological differences in race continue to shape the way physicians perceive and treat Black patients and exacerbate racial disparities in treatment and outcomes, and real change must be structural and multi-pronged.

## Discussion

Reparations are a way to make amends for wrongs or injuries inflicted. The impact of racism found within healthcare in terms of differential morbidity and mortality is more than enough to justify reparations to the Black community (37). Reparations must satisfy two criteria: first, they must seek to rectify past injustices of specific populations. Second, they must remedy current inequitable outcomes. Medicaid is a powerful intervention that helps many historically marginalized populations, but based on the two aforementioned criteria, it would not count as a form of reparations. This is because Medicaid is technically available to all people of a certain socioeconomic status, and it does not specifically seek to remedy current inequities due to historical and transgenerational trauma. Medicine's historical role in presenting mythical uses of racial categories to justify racism—which fueled the inequities in criminal justice, housing, education, and other systems today—requires immediate and long overdue action.

While reparations typically refer to financial compensation, reparative policies need not solely consist of direct monetary transfers. Rather, reparative policies can be those accomplishing two things: first, they break down existing processes that maintain the racial hierarchy by empowering the individuals they oppress. Second, their aim is to achieve equity in outcomes, not just equality. Although both equity and equality attempt to promote justice, equality achieves this through treating everyone the same regardless of need, whereas equity achieves this through treating people differently depending on need. Therefore, a health inequity is a difference in a health outcome between population cohorts caused by avoidable systemic structures rooted in racial, social, environmental, or economic injustice.

We suggest that the development of reparative policies in healthcare be guided by a framework such as the Critical Race Public Health Praxis (CRPHP) (38, 39). CRPHP highlights the need to honor the voice of socially marginalized groups, address structural determinism, and engage in disciplinary self-critique. For each of these principles, we suggest examples of policies that embody them.

First, to honor the voice of marginalized groups, reparative policies must be shaped by the communities in which they are trying to serve. In other words, the community voice must be operationalized into interventions to ensure needs are appropriately understood and met. For example, in the South Africa's Pholela Community Health Center model, and the related Community Health Center model in the United States facilitated by Jack Geiger, 50% of the leadership had to be people who use the health care system (40). In the 1960s and 1970s, the Black Panther Party created a Breakfast Program to feed thousands of Black children. This program expanded to 45 other similar initiatives to cover everything from free medical clinics to community ambulance services and legal clinics (41). In these examples, the policies were enacted by the community they were designed to serve. Of note, community health centers are not without their limitations, such as how “community” is vaguely defined and whether decentralization might breed inefficiency in care (42, 43). Nevertheless, the goal of reparative policies is to restructure the way in which decisions in healthcare are made so that those in the community context—who would be most affected by the policies—are the primary decision-makers. Creating free, community-led health clinics would elevate equitable access and treatment—as well as provide sustainable medical infrastructure—in marginalized communities.

Second, to address structural determinism, reparative policies must be race-conscious rather than race-blind (44). Indeed, there is a rich literature demonstrating the impact of structural racism on the health of Black Americans (45). For example, when adjusting for other sociodemographic factors, being Black is independently associated with increased incidence of mental health conditions such as anxiety and depression, as well as hypertension. Whereas these differences have been assumed to be due to genetic differences, research shows that they are not due to innate biological differences but rather influences of chronic and unremitting stress caused by racism (46). Therefore, reparative policies must be race-conscious and deliberatively seek to benefit Black and non-White populations. Colorblind policies would not lead to more equity. In fact, evidence shows that colorblindness upholds the racial status quo and inhibits efforts to promote health equity (47). While healthcare has worked hard to rid our discourse from negative biases, stereotypes, and discriminatory language that adversely impact people of color, we need to add back to our discourse the notion of race and identity (48). We must see and acknowledge race, and thereby acknowledge the harms of racism. In doing so, we can fully account for the adverse effects of race and racism on health.

A specific reparative policy that is race-conscious rather than race-blind and addresses structural determinism is payment reform, such as the expansion of Medicaid coverage or new, state-specific health insurance for non-White populations. For example, pulmonary rehabilitation has been found to be an effective treatment option for those with COPD (49). Yet, since not all insurance carriers cover it, Whites disproportionately benefit. This has led to disparities in COPD and other respiratory treatments, which are likely to be exacerbated during the COVID-19 pandemic. Reforming payment for effective treatments for diseases in addition to COPD that are more prevalent in minority populations, such as diabetes and heart disease, would also achieve greater justice (50).

Finally, to engage in disciplinary self-critique, the fields of clinical medicine and research science should adopt certain reparative policies that embody the same themes and change the process by which decisions are made to shatter the status quo racial hierarchy. Clinical medicine interventions may include mandating voices of historically marginalized voices in the governance of hospitals that receive public funds, funding for community-driven interventions to address social determinants of health tied to historically racist policies (e.g., housing voucher programs, food programs), and pipeline programs to recruit young people from communities of color into health careers. So long as policies seek to rectify past injustices of specific populations as well as remedy current inequitable outcomes, regardless of whether there is a financial component, they are reparative. These are necessary but not sufficient by themselves to righting past wrongs and achieving greater health equity.

Research science has also played an important and unique historical role in developing the architecture of racism in America. The central racism in the conception and execution of research science manifested in J. Marion Sims's experiments in obstetrics to the use of Henrietta Lacks's stem cells for research without her consent. Thus, research science itself must face a reparative reckoning. After the unconscionable experiments on vulnerable groups during World War II by German and Japanese scientists, ultimately, research science in the United States took the dramatic step of embracing and operationalizing (via Institutional Review Boards) a code of ethics that was more protective of the rights of individuals. There is no reason that half a century later, a similar, significant reform, centering and operationalizing racial equity and community engagement would not be possible. Reparative justice in research might include forcing more diversity in research subjects (away from White males) and ensuring clinical subjects and target populations are guaranteed continuation of clinical effective treatments.

While healthcare is crucial to the discussion since it paved the foundation for racism in all other aspects of daily life, broader concerns relating to housing, jobs, law, and the like deserves attention. Structural racism in these other contexts must be tackled, lest any advances in health equity ultimately be moot. The specifics of how changes in these areas might be achieved is beyond the scope of this paper, but examples include eliminating food deserts from particular minority-heavy neighborhoods; altering housing laws to encourage more rent-controlled subsidies and affordable house ownership; and reforming the criminal justice system away from antiquated laws from the War on Drugs during the Nixon Administration that disproportionately affect communities of color (51). Addressing systemic racism in all fields is vital to ensure that advances in health equity are optimized and sustainable.

How will we know the need for reparative policies is over? There are many considerations, but policies first and foremost should be tied to data-driven outcomes. Reparations may no longer be needed once there is equity in health outcomes between different races for similar diseases and conditions. Next to quantitative metrics, subjective measures can drive reparative policies, measures such as the level and restoration of trust in medicine by the Black community. Ultimately, the decision of when to start and stop reparations must be iterative, with ongoing considerations and reviews. Finally, we want to re-emphasize that while this piece focused on reparative policies for Black Americans based on the medical profession's history of racism, there are other historically marginalized populations, such as the Native American and other communities of color, that have suffered unmeasurable historical injustice, including specifically at the hands of physicians (52), and they must also be the beneficiaries of similar, necessary reparative policies.

In conclusion, the inequitable morbidity and mortality as a result of COVID-19 across racial groups in the United States demonstrates that historical racism in healthcare, housing, employment, and education continues to shape society. Now, more than ever, health reparations are needed. It was racist physicians and scientists who fabricated the racial hierarchy based on fabricated scholarship that people of different races hold biologically different statuses, a belief used to maintain the power of Whiteness. Therefore, the healthcare profession has an ethical duty to pursue health-related policies designed to rectify past injustices and remedy current inequitable outcomes related to historical and systemic racism. While our list of examples could never be complete, a principle-based approach that seeks to amplify the community voice, address structural determinism, and engage in self-critique and historical reawakening is fundamental to a more equitable future. Indeed, the proposed health reparations would not be sufficient to create more equitable health outcomes, but they will foster an equitable health system built on justice for all, especially society's most vulnerable.

## Data Availability Statement

The original contributions presented in the study are included in the article/supplementary material, further inquiries can be directed to the corresponding author/s.

## Author Contributions

DS conceptualized the idea and drafted the manuscript. AC, DO, and EL all participated in edits and revisions to the manuscript. All authors reviewed and approved the final version of the manuscript.

## Conflict of Interest

The authors declare that the research was conducted in the absence of any commercial or financial relationships that could be construed as a potential conflict of interest.
